# Cryoballoon Ablation Strategy in Persistent Atrial Fibrillation

**DOI:** 10.3389/fcvm.2021.758408

**Published:** 2021-11-18

**Authors:** Florian Straube, Janis Pongratz, Alexander Kosmalla, Benedikt Brueck, Lukas Riess, Stefan Hartl, Christian Tesche, Ullrich Ebersberger, Michael Wankerl, Uwe Dorwarth, Ellen Hoffmann

**Affiliations:** ^1^Department of Cardiology and Internal Intensive Care Medicine, Heart Center Munich-Bogenhausen, Munich Clinic Bogenhausen, Academic Teaching Hospital of the Technical University Munich, Munich, Germany; ^2^Faculty Munich University Clinic, Ludwig-Maximilians-University, Munich, Germany; ^3^KardiologieErkelenz, Erkelenz, Germany; ^4^Cardiology, University of Düsseldorf, Düsseldorf, Germany; ^5^Department of Cardiology, Klinik Augustinum, Munich, Germany; ^6^KMN—Kardiologie Muenchen Nord, Munich, Germany

**Keywords:** atrial fibrillation, catheter ablation, cryoballoon ablation, pulmonary vein isolation, persistent atrial fibrillation

## Abstract

**Background:** Cryoballoon ablation is established for pulmonary vein isolation (PVI) in paroxysmal atrial fibrillation (AF). The objective was to evaluate CBA strategy in consecutive patients with persistent AF in the initial AF ablation procedure.

**Material and Methods:** Prospectively, patients with symptomatic persistent AF scheduled for AF ablation all underwent cryoballoon PVI. Technical enhancements, laboratory management, safety, single-procedure outcome, predictors of recurrence, and durability of PVI were evaluated.

**Results:** From 2007 to 2020, a total of 1,140 patients with persistent AF, median age 68 years, underwent cryoballoon ablation (CBA). Median left atrial (LA) diameter was 45 mm (interquantile range, IQR, 8), and Congestive heart failure, Hypertension, Age ≥75 years (doubled), Diabetes mellitus, prior Stroke or TIA or thromboembolism (doubled), Vascular disease, Age 65 to 74 years, Sex category (CHA_2_DS_2_-VASc) score was 3. Acute isolation was achieved in 99.6% of the pulmonary veins by CBA. Median LA time and median dose area product decreased significantly over time (*p* < 0.001). Major complications occurred in 17 (1.5%) patients including 2 (0.2%) stroke/transitory ischemic attack (TIA), 1 (0.1%) tamponade, relevant groin complications, 1 (0.1%) significant ASD, and 4 (0.4%) persistent phrenic nerve palsy (PNP). Transient PNP occurred in 66 (5.5%) patients. No atrio-esophageal fistula was documented. Five deaths (0.4%), unrelated to the procedure, occurred very late during follow-up. After initial CBA, arrhythmia recurrences occurred in 46.6% of the patients. Freedom from atrial arrhythmias at 1-, and 2-year was 81.8 and 61.7%, respectively. Independent predictors of recurrence were LA diameter, female sex, and use of the first cryoballoon generation. Repeat ablations due to recurrences were performed in 268 (23.5%) of the 1,140 patients. No pulmonary vein (PV) reconduction was found in 49.6% of the patients and 73.5% of PVs. This rate increased to 66.4% of the patients and 88% of PVs if an advanced cryoballoon was used in the first AF ablation procedure.

**Conclusion:** Cryoballoon ablation for symptomatic persistent AF is a reasonable strategy in the initial AF ablation procedure.

## Introduction

In symptomatic persistent atrial fibrillation (AF), catheter ablation is an established treatment option (1). Pulmonary vein isolation (PVI) is the cornerstone of AF ablation and is recommended with a class I, level of evidence A in the current guideline (2). Additional ablation procedures beyond PVI are not well-established; therefore, current guidelines give a class IIb recommendation (1, 2).

Cryoballoon ablation is a standard treatment procedure for symptomatic paroxysmal AF (1) and has become a first-line treatment for this type of symptomatic AF recently (3–5). Cryoballoon ablation (CBA) for symptomatic persistent AF has been evaluated in a pilot study with the less effective first generation cryoballoon (CBG1): A double balloon strategy with the concomitant treatment with both, the 23 and 28 mm cryoballoon aiming at broad and wide circumferential antral ablation demonstrated favorable initial results (6). After the introduction of the more effective second-generation cryoballoon (CBG2) in 2012, the single big balloon strategy became an option for PVI in persistent AF.

A CBA system (Arctic Front™; Cryocath Inc.) was approved by the European Union (EU) for the interventional treatment of AF in 2005, and the Food and Drug Administration (FDA) approved the system in 2010 (Arctic Front™, Medtronic Inc.) to treat drug-refractory paroxysmal AF. Recently, in June 2020, the FDA extended the labeling for the treatment of drug-refractory recurrent symptomatic persistent AF (episode duration <6 months) mainly based on the results of the CRYO4PERSISTENT single-arm trial (7): A total of 101 patients with early persistent AF were treated by means of CBA. Single-procedure success was 61% at 12 months post-ablation in addition to significant reduction in arrhythmia-related symptoms and improved quality of life (7).

In 2018, Omran et al. included 917 patients with persistent AF from 11 CBA studies into a meta-analysis. Freedom from AF was 68.9% at 16.7 months (8). Most of the studies were observational, and selected patients were included. The number of patients was limited in all of the studies (≤ 180 patients).

The objective of this large all-comer study was to evaluate and share our experience with CBA as the exclusive ablation strategy in consecutive patients for the initial ablation procedure in symptomatic persistent AF. Efficacy, safety, and information on the durability of PVI from repeat procedures were evaluated.

## Materials and Methods

Consecutive patients with symptomatic persistent AF scheduled for the first AF ablation procedure were prospectively included into the observational single-center study. All the patients were treated by means of CBA. Exclusion criteria were according to the guidelines. Patients with LA diameter > 60 mm were not referred for AF ablation. Patients with long-standing persistent AF were pretreated with antiarrhythmic drugs, and sinus rhythm was established by cardioversion. Cessation of anti-arrhythmic drug (AAD) treatment was recommended after the procedure.

Procedural and periprocedural results, complications, and the outcome were documented. The primary endpoint was freedom from any atrial arrhythmia recurrences, AF or atrial tachycardia (AT), and off antiarrhythmic drugs following a 90-day blanking period. Patients with AAD at follow-up were calculated as failures after the blanking period. Relevant typical AF symptoms > 30 s reported by the patients without any ECG documentation were calculated as a recurrence.

### Cryoballoon Procedure

The procedure has been described in detail in previous studies (6, 9–14). Prior to the procedure, baseline characteristics were documented, and all the patients underwent transthoracic and transesophageal echocardiography.

The procedure was performed under conscious sedation. After venous access, single transseptal puncture was performed with a guiding sheath and BRK needle and was guided by transesophageal or intracardiac echocardiography. A steerable sheath (Flexcath or Flexcath Advance™; Medtronic Inc., Minneapolis, MN, United States) was positioned in the left atrium over a guidewire. Four different cryoballoon generations were used depending on its availability in the study: Arctic Front™ (CBG1), Arctic Front Advance™ (CBG2), Arctic Front Advance ST™ (CBG3), and Arctic Front Advance Pro™ (CBG4), which were all from Medtronic Inc. (Minneapolis, MN, United States). The cryoballoon was introduced over a stiff wire or the spiral mapping catheter (SMC) and positioned at the ostium of the PV. Three different SMCs with different sizes were available during the inclusion period: a 6-pole mapping catheter with a 15-mm loop (ProMap™; ProRhythm Inc., Ronkonkoma, NY, United States), or an 8-pole with 15- or 20-mm loop (Achieve™ or Achieve Advance™), or a 10-pole with 25-mm loop (Achieve Advance™) (the latter are all from Medtronic Inc., Minneapolis, MN, United States). ACT was kept between 300 and 400 s with unfractionated heparin. PV potentials were recorded at least before and after complete PVI with a circular mapping catheter. Periprocedural management was performed in accordance with the current practice guidelines (2). Balloon positioning was visualized by fluoroscopy and intracardiac echocardiography if available. Immediately before the start of the cryoablation cycle, occlusion was determined with a contrast injection through the inner lumen of the cryoballoon.

**Figure 1:**
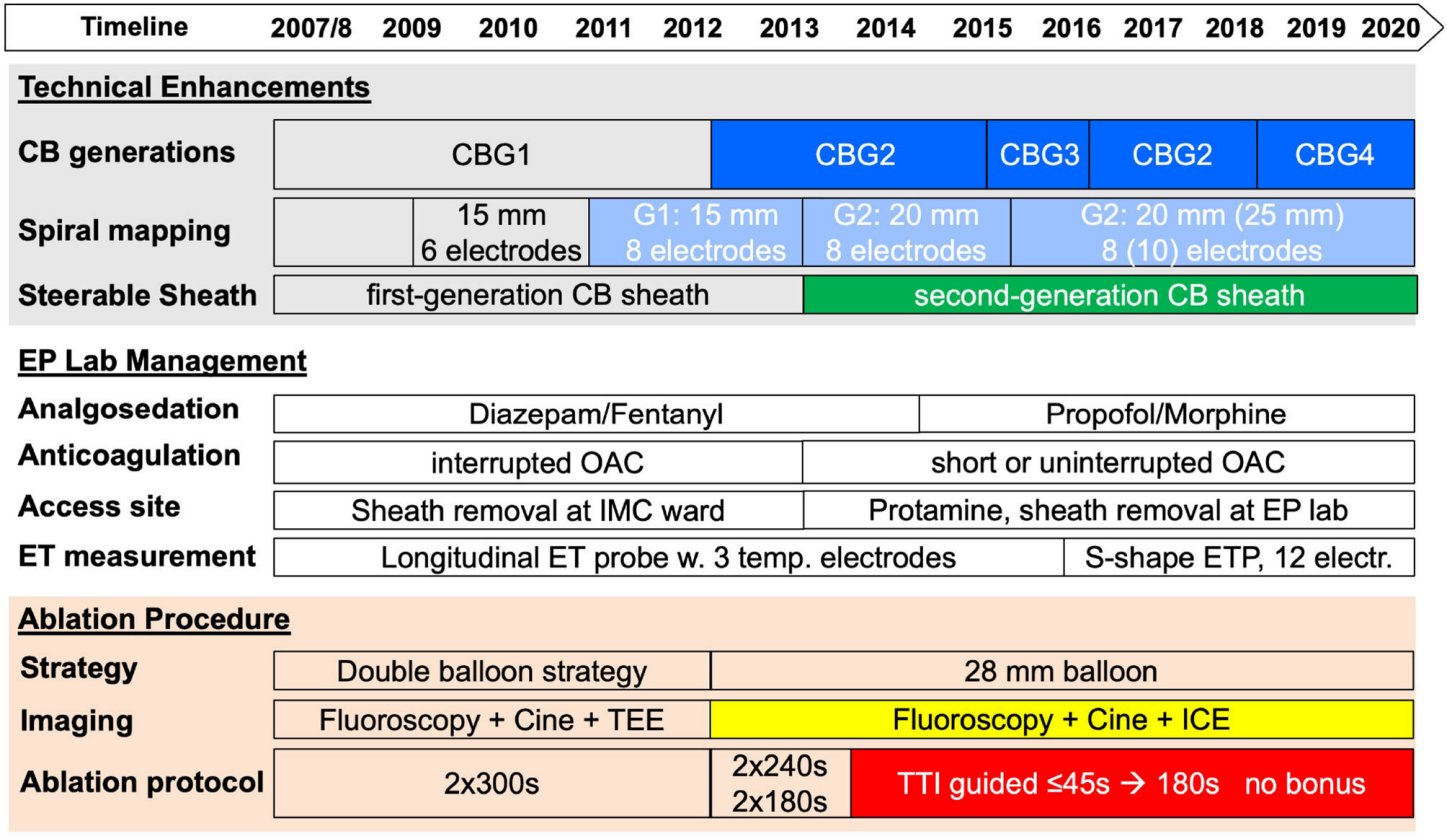
Evolution of cryoballoon ablation for persistent atrial fibrillation (AF) from 2007 to 2020. A timeline of technical enhancements, improvement in laboratory management, and implementation of new cryoballoon protocols are shown in the upper part of the figure. CB, cryoballoon; CBG, cryoballoon generation; EP, electrophysiology; ET, esophageal temperature; ICE, intracardiac echocardiography; TEE, transesophageal echocardiography; TTI, time-to-isolation.

With the introduction of the CBG1, with an equatorial cooling zone, we applied a double balloon strategy as part of a persistent AF proof of concept study using both the 23- and 28-mm balloons aiming at optimized PV occlusion and isolation with the smaller balloon followed by a wide circumferential lesion induction with the larger balloon. With the availability of the more effective CBG2 (broader cooling zone of the distal hemisphere of the balloon, increased refrigerant flow rate), the strategy was adapted to the primary use of the 28-mm balloon as the catheter. In case of inability to isolate the PV with the described setting, it was at the discretion of the operator to change the SMC to a stiff wire, use the small 23 mm cryoballoon only in PVs with an ostial maximum diameter of ≤ 21 mm, or use RF touch-up applications to isolate the PV. The institutional protocol for all cryoballoon ablation procedures evolved from 2007 to 2020 (Figure 1).

An endoluminal esophageal linear (SensiTherm; St. Jude Medical, Saint Paul, MN, United States) or sinusoidal temperature probe with 12 thermocouples (CIRCA S-Cath™; CIRCA Scientific, Englewood, CO, United States) was used in all the patients. Phrenic nerve (PN) stimulation was controlled by palpation and intracardiac echocardiography visualization of the diaphragmatic motion if available. For premature termination due to esophageal temperatures ≤ +15°C, PN impairment or balloon temperatures ≤ −55°C for the right superior PV, double stop technique was applied after its introduction in 2012/2013. See details for the evolution of the modification of the ablation protocol in Figure 1.

### Post-procedure Care

Transthoracic echocardiography was performed in all the patients to rule out pericardial effusion. From 2007 to 2013, sheath removal was performed in the intermediate care ward after activated clotting time decreased to ≤ 200 s. Since 2013, protamine has been administered immediately before sheath removal in the lab. From 2007 to 2013, vitamin K antagonists (VKAs) were interrupted up to 7 days before the intervention, and bridging with low-molecular weight heparin was performed. Intravenous heparin was used after the procedure until the next day. Low molecular-weight heparin was administered to patients on VKAs with INR <2 until therapeutic INR 2–3 was achieved. Since 2013, in patients on VKAs with an INR of 2 to 3, the procedure has been carried out without discontinuation of the oral anticoagulation. In patients on novel oral anticoagulants, the intake of a novel oral anticoagulant in the morning was postponed until after the procedure. Novel oral anticoagulants were reinitiated after hemostasis and no later than 1 h after ablation. Anticoagulation was continued for ≥ 3 months, and thereafter based on individual CHA_2_DS_2_-VASc score. All the patients were empirically treated with proton-pump inhibitors for 4 weeks.

### Study Follow-Up

All the patients were monitored during the hospital stay for at least 24–48 h post intervention, including continuous monitoring in the intermediate care ward, by telemetry and/or 24–72-h Holter studies.

Routine follow-up included symptom evaluation, 12-lead ECG, and Holter studies (1–7 days). Follow-up was performed by the outpatient clinic in collaboration with referring physicians and by structured telephone interview. In patients with repeat ablation following CBA due to recurrence, data on the durability of PVI were documented prospectively.

### Statistical Analysis

In accordance with the Shapiro–Wilk test, continuous variables are expressed as means with standard deviation (SD) or as medians with quartiles. Categorical data are shown as numbers and percentages and are compared by the chi-square test. Friedman's two-way analysis of variance by Ranks was performed to test for differences between groups, if more than two groups with non-normal distribution were compared. Kaplan–Meier Survival Curve for the endpoint freedom from any atrial arrhythmia recurrence was calculated, and log-rank test was performed for comparison. Univariate Cox regression analyses were performed to identify predictors of recurrence. Main independent risk factors were determined using a stepwise multivariate Cox regression model with bidirectional elimination including only parameters of highest univariate significance. Database management system FileMaker Pro (Claris International Inc., Santa Clara, CA, United States) was used. Data processing and analysis were performed using Excel 2010 (Microsoft Corp., Redmond, WA, United States) and SPSS 20.0 (IBM Corp., Armonk, NY, United States). Statistical significance was defined as *p* ≤ 0.05.

## Results

### Study Population and Differences Among the Treatment Groups

From 2007 to 2020, 1,140 consecutive patients with symptomatic persistent atrial fibrillation underwent AF ablation at the Heart Center Munich-Bogenhausen. All of them underwent electrical disconnection of the pulmonary veins by means of CBA. The procedures were performed by five experienced operators. Four different cryoballoon generations were available over time (Figure 1), and treatment groups were created accordingly. Baseline characteristics were different in the treatment groups. Older age, more females, larger LA diameter sizes, lower ejection fraction, more hypertension and hypertensive heart disease, more coronary heart disease and cardiomyopathies were observed in the treatment groups with the advanced cryoballoon catheters (CBG2, CBG3, and CBG4) as compared with CBG1. A significant decline of the body mass index was observed over time (see Table 1 for details). No significant difference was observed for the finding of the left common ostium, but significantly more right accessory PVs were documented with the use of CBG1.

**Table 1:** Baseline characteristics and differences in the treatment groups.

	**Total**	**CBG1**	**CBG2**	**CBG3**	**CBG4**	***p*-value**
N patients (%)	1,140 (100)	208 (18.2)	613 (53.8)	112 (9.8)	207 (18.2)	
Age, years	68 [15]	63 [13]	69 [15]	69 [17]	69 [16]	** <0.001**
Females (%)	391 (34.3)	49 (23.6)	232 (35.7)	40 (35.7)	70 (33.8)	**0.003**
LA diameter^*^ mm	45 [8]	44 [5]	45 [8]	44 [6]	44 [8]	**0.001**
Ejection fraction%	55 [10]	60 [8]	55 [10]	55 [5]	55 (9)	** <0.001**
AAD I/III prior to the procedure (%)	310/465 (66.7)	15/22 (68.2)	170/242 (70.2)	19/28 (67.9)	106/173 (61.3)	0.30
Number of electrical cardioversions	2 [2] *n* = 521	n/a	2 [2] *n* =283	2 [2] *n* = 30	2 [2] *n* = 207	0.09
Number of Episodes per year	3 [3] *n* = 470	n/a	3 [4] *n* = 254	3 [3] *n* = 30	3 [3] *n* = 185	0.97
Max. duration of a single AF episode, days	30 [80] *n* = 460	n/a	30 [80] *n* = 258	7 [21] *n* = 29	30 [75] *n* = 172	**0.001**
Hypertension	819 (73.3)	130 (62.5)	455 (76.1)	78 (75.0)	156 (75.4)	**0.002**
Hypertensive heart disease	403 (36.1)	38 (18.3)	225 (37.6)	50 (48.1)	90 (43.7)	** <0.001**
Mitral regurgitation ≥ II	61 (5.4)	6 (2.9)	37 (6.0)	4 (3.6)	14 (6.8)	0.20
Coronary artery disease	283 (24.8)	32 (15.4)	149 (24.3)	28 (25.0)	74 (35.7)	** <0.001**
Prior myocardial infarction	36 (3.2)	3 (1.4)	19 (3.2)	4 (3.8)	10 (4.9)	0.26
Cardiomyopathy	148 (13.0)	13 (6.3)	78 (12.7)	19 (17.0)	38 (18.4)	**0.002**
Structural heart disease	334 (29.7)	86 (41.3)	175 (19.2)	62 (55.4)	11 (5.4)	** <0.001**
Diabetes mellitus	41/543 (7.6)	3/24 (12.5)	23/284 (8.1)	1/30 (3.3)	14/205 (6.8)	0.60
CHA_2_DS_2_-VASc Score	3 [2] *n* = 486	n/a	3 [2] *n* = 263	3 [2] *n* = 28	3 [2] *n* = 194	0.61
BMI kg/m^2^	27.5 [6.1]	28.6 [7.8]	27.1 [5.9]	26.4 [4.2]	26.6 [6.5]	**0.008**
Overweight (BMI >25)	593 (52.5)	63 (30.3)	350 (57.1)	49 (43.8)	131 (63.3)	** <0.001**
Obesity (BMI >30)	242 (21.2)	30 (14.4)	140 (22.8)	16 (14.3)	56 (27.1)	**0.003**
Obesity °II/III (BMI >35)	75 (6.6)	14 (6.7)	42 (6.9)	3 (2.7)	16 (7.7)	0.35
Common ostium	88 (7.7)	11 (5.3)	56 (9.1)	11 (9.8)	10 (4.8)	0.09
Accessory veins	51 (4.5)	17 (8.2)	29 (4.7)	4 (3.6)	1 (0.5)	**0.002**

*N (%): number of patients and percentage; mean ± standard deviation or median with interquartile range [in squared brackets] as appropriate according to the test of normal distribution*.

*AAD, anti-arrhythmic drug; AF, atrial fibrillation; BMI, body mass index; CBG, cryoballoon generation; LA, left atrium; CHA2DS2-VASc, Congestive heart failure, Hypertension, Age ≥75 years (doubled), Diabetes mellitus, prior Stroke or TIA or thromboembolism (doubled), Vascular disease, Age 65 to 74 years, Sex category*.

* *determined by echocardiography (anterior-posterior diameter)*.

*Bold values are referred statistically significant*.

### Advances in CBA for Persistent AF

The introduction of the second cryoballoon generation in 2012 with an increased refrigerant flow rate and a broader cooling zone led to a continuous adaption of the ablation protocol. The reduction of freezing time per application, implementation of time-to-isolation guided ablation protocols, omission of the double balloon strategy, and omission of the routine bonus application became possible. In addition to the learning curve of the operators over more than a decade, lab management was optimized by the implementation of modern analgo-sedation, optimized access site management, advanced esophageal temperature monitoring, and use of intracardiac echocardiography. Details are presented in Figure 1.

### Acute Efficacy and Effectiveness

**Figure 2:**
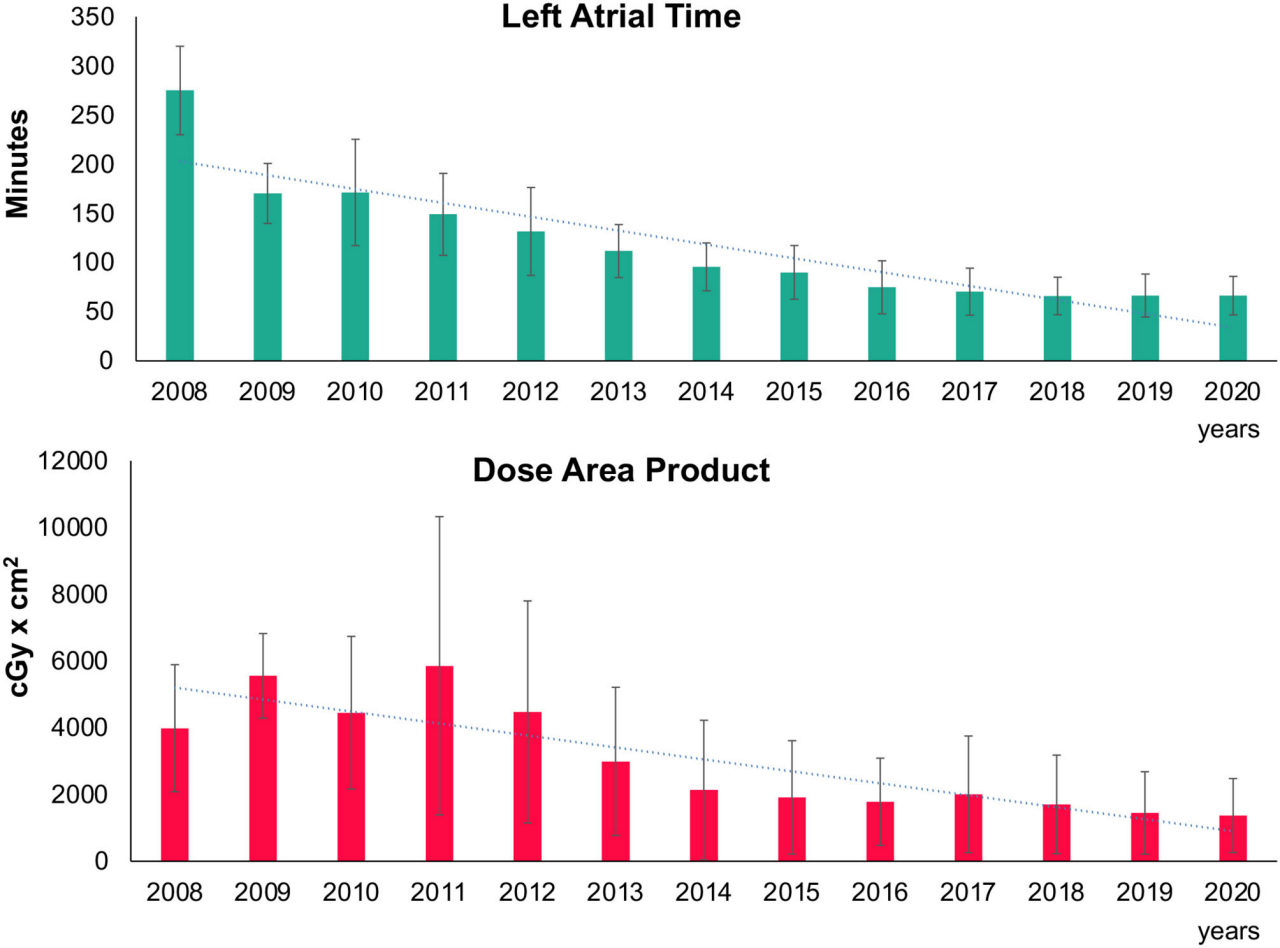
Trend in left atrial procedure time and radiation. Mean left atrial procedure time (upper part) and dose area product (lower part) with standard deviation are depicted in bar charts over time. Trend line shows the decline over the years.

The acute efficacy measured by the rate of isolated PV at the end of the procedure increased within the treatment groups ranged from 99.1 to 100%, *p* = 0.011. After the introduction of the advanced cryoballoons (Arctic Front Advance™/Advance ST™/Advance Pro™; all from Medtronic Inc.), the double balloon rate decreased significantly (*p* < 0.001). A continuous and statistically significant decrease in LA dwelling time (Figure 2), fluoroscopy time, and dose area product (Figure 2), which represents the radiation risk from the interventional procedure, were found (see Table 2 for details).

**Table 2:** Procedural characteristics and differences in the treatment groups.

	**Total**	**CBG1**	**CBG2**	**CBG3**	**CBG4**	***p*-value**
N (%)	1,140 (100)	208 (18.2)	613 (53.8)	112 (9.8)	207 (18.2)	** <0.001**
Total veins	4,378 (100)	819 (100)	2,331 (100)	409 (100)	819 (100)	
Acute PVI	4,360 (99.6)	812 (99.1)	2,324 (99.7)	405 (99.0)	819 (100)	**0.011**
28 mm CB %	812 (71.2)	46 (22.1)	468 (76.3)	95 (84.8)	203 (98.1)	** <0.001**
23 mm CB %	63 (5.5)	28 (13.5)	35 (5.7)	0 (0)	0 (0)	** <0.001**
Both balloons %	265 (23.2)	134 (64.4)	110 (17.9)	17 (15.2)	4 (1.9)	** <0.001**
LA Time min.	80 [57]	145 [50]	80 [45]	80 [30]	60 [20]	** <0.001**
Fluoroscopy Time min.	26 [17]	36 [18]	19 [12]	20 [11]	14.5 [7]	** <0.001**
Dose area product cGyxcm^2^	1,818 [2,578]	4,184 [3,793]	1,704 [2,126]	1,433 [1,477]	1,129 [1,259]	** <0.001**

*N (%): number of patients and percentage; mean ± standard deviation or median with interquartile range [in squared brackets] as appropriate according to the test of normal distribution*.

*CB, cryoballoon; CBG, cryoballoon generation; LA, left atrium; PVI, pulmonary vein isolation; cGy: centigray, cm: centimeter*.

*Bold values are referred statistically significant*.

### Safety

The rate of adverse events following the procedure is presented in Table 3 for the total population and treatment groups. No procedure-related death and no atrioesophageal fistula were observed. Five deaths (0.4%) unrelated to the procedure occurred very late during long-term follow-up. The major complication rate was 1.5% in the total population, and no difference was observed among the treatment groups. Pericardial tamponade was observed in 1 of the 1,140 procedures (0.09%). The rate of thromboembolic cerebrovascular complications was 0.2%. Minor adverse events were observed in 8.9% of the patients, and included 5.3% transient phrenic nerve palsy (PNP), which recovered completely until discharge of the patients. The rate of transient PNP decreased significantly over time. The rate of persistent phrenic nerve palsy was 0.4%. Pulmonary infections were infrequent after the CBA, and the rate declined significantly among the treatment groups.

**Table 3:** Complications and differences in the treatment groups.

	**Total**	**CBG1**	**CBG2**	**CBG3**	**CBG4**	***p*-value**
	1,140 (100)	208 (18.2)	613 (53.8)	112 (9.8)	207 (18.2)	
Total major complications	17 (1.5)	4 (1.9)	9 (1.5)	1 (0.9)	3 (1.4)	0.95
Deaths	0 (0)	0 (0)	0 (0)	0 (0)	0 (0)	1.00
Myocardial infarction	0 (0)	0 (0)	0 (0)	0 (0)	0 (0)	1.00
TIA/stroke	2 (0.2)	0 (0)	2 (0.3)	0 (0)	0 (0)	0.63
Pericardial tamponade	1 (0.1)	0 (0)	1 (0.1)	0 (0)	0 (0)	0.83
PV stenosis	0 (0)	0 (0)	0 (0)	0 (0)	0 (0)	1.00
Groin complications with surgery/intervention	9 (0.8)	4 (1.9)	3 (0.5)	1 (1.0)	1 (0.5)	0.54
Arial septal defect treated by intervention	1 (0.1)	0 (0)	1 (0.1)	0 (0)	0 (0)	0.83
Unresolved phrenic nerve palsy until discharge	4 (0.4)	0 (0)	2 (0.3)	0 (0)	2 (1.0)	0.36
Total minor complications	98 (8.6)	45 (21.6)	38 (6.2)	5 (4.5)	10 (4.8)	** <0.001**
Transient phrenic nerve palsy	60 (5.3)	31 (14.9)	24 (3.9)	1 (0.9)	4 (1.9)	** <0.001**
Pulmonary infection	6 (0.5)	5 (2.4)	0 (0)	0 (0)	1 (0.5)	**0.001**
Pericardial effusion	3 (0.3)	1 (0.5)	2 (0.3)	0 (0)	0 (0)	0.73
Others	29 (2.5)	8 (3.8)	12 (2.0)	4 (3.6)	5 (2.4)	0.66
Total	115 (10.1)	49 (23.6)	46 (7.5)	6 (5.4)	13 (6.3)	** <0.001**

*N (%): number of patients and percentage. CBG, cryoballoon generation; TIA, transitory ischemic attack; PV, pulmonary vein*.

*Bold values are referred statistically significant*.

The change of the analgo-sedation regime from intermittent diazepam and fentanyl application to continuous propofol and intermittent morphine application, and the implementation of uninterrupted or shortly interrupted oral anticoagulation were both not associated with more complications.

### Outcome of CBA in Persistent AF

After a single procedure and a median follow-up time of 22 months, atrial arrhythmia recurrences, defined as AF, atrial tachycardia, or atypical atrial flutter occurred in 520 (45.6%) of the 1,140 patients. Kaplan–Meier estimates for arrhythmia free survival were 81.8% at 1 year, and 61.7% at 2 years of follow-up.

**Figure 3:**
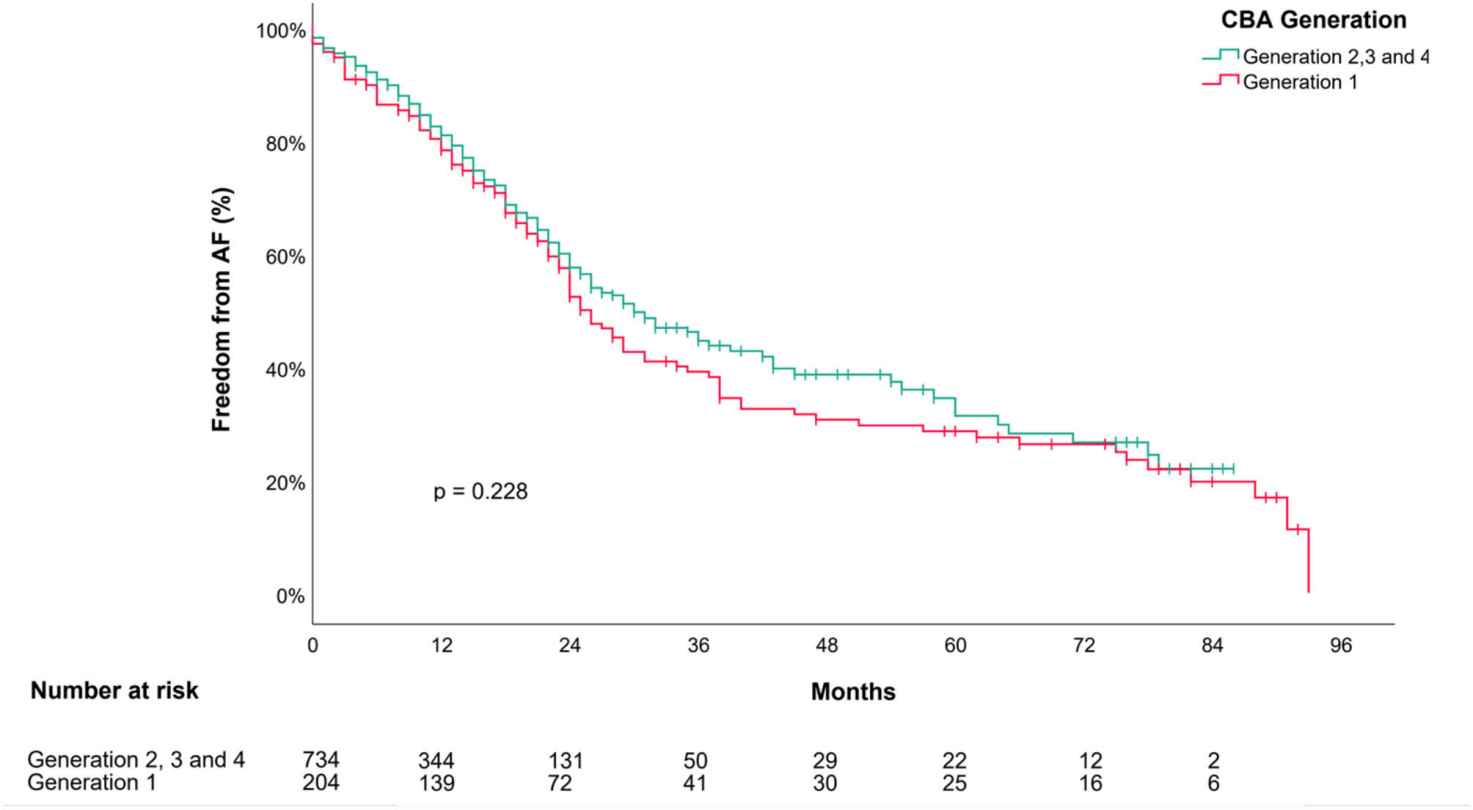
Outcome of cryoballoon ablation in persistent AF after a single procedure. Kaplan–Meier Survival Curve depicts estimates for “Freedom from AF/AT” after a single cryoballoon ablation stratified following the first (CBG1; in red color) or an advanced cryoballoon generation (CBG2, CBG3, or CBG4; in Turquoise color) of the balloon. *P*-value is provided for the log-rank comparison of the curves. AF, atrial fibrillation; CBA, cryoballoon ablation.

The Kaplan–Meier Curves in Figure 3 show the unadjusted outcome estimates according to the treatment with the first- or the next-generation cryoballoon. No difference was observed among the groups (log-rank test, *p* = 0.22).

### Predictors of Recurrence

After adjustment for baseline characteristics and treatment groups, independent risk factors for recurrence were female sex (adjusted HR 1.42, 95% CI 1.114–1.798; *p* = 0.004) and LA diameter determined by transthoracic echocardiography (adjusted HR 1.03, 95% CI 1.014–1.048, *p* = 0.001). The relative risk of recurrence increased by 3% per millimeter of the LA diameter. The use of the advanced cryoballoon generations was an independent predictor for success (adjusted HR 0.77, 95% CI 0.605–0.976). See Table 4 for details.

**Table 4:** Predictors of outcome: uni- and multivariate analyses.

	**Univariate analysis**			**Multivariate analysis**		
	**HR**	**95% CI**	***p*-value**	**HR**	**95% CI**	***p*-value**
Age	1.00	0.996–1.016	0.21	1.00	0.991–1.014	0.65
Female	1.29	1.039–1.593	**0.02**	1.42	1.114–1.798	**0.004**
LA Diameter	1.06	1.009–1.042	**0.002**	1.03	1.014–1.048	**0.001**
Ejection fraction	1.00	0.982–1.009	0.50			
AAD I/III prior to the procedure	1.17	0.753–1.822	0.48			
Number of electrical cardioversions	1.00	0.886–1.149	0.887			
Number of episodes per year	1.00	0.999–1.005	0.22			
Max. duration of episodes	1.00	0.999–1.002	0.40			
Hypertension	0.98	0.779–1.235	0.87			
Hypertensive heart disease	0.96	0.768–1.209	0.78			
Mitral regurgitation ≥°II	1.17	0.744–1.837	0.49	1.15	0.719–1.829	0.57
Coronary artery disease	0.80	0.620–1.054	0.12			
Prior myocardial infarction	0.66	0.296–1.487	0.32			
Cardiomyopathy	1.10	0.775–1.547	0.61			
Structural heart disease	1.16	0.937–1.430	0.17			
Diabetes mellitus	0.98	0.455–2.124	0.97			
CHA_2_DS_2_-VASc	0.16	0.959–1.297	0.16			
Body mass index	1.00	0.972–1.027	0.94			
Overweight (BMI >25)	0.87	0.713–1.077	0.21			
Obesity (BMI >30)	0.97	0.742–1.262	0.80			
Obesity °II/III (BMI >35)	1.35	0.915–1.984	0.13	1.23	0.812–1.868	0.33
Common Ostium	1.06	0.731–1.527	0.77			
Accessory Veins	1.12	0.727–1.723	0.61			
Use of an advanced cryoballoon catheter^*^	0.87	0.700–1.091	0.23	0.77	0.605–0.976	**0.03**
Only 23 mm	0.97	0.670–1.411	0.83			
Only 28 mm	0.56	0.763–1.159	0.56			
23 + 28 mm	1.08	0.869–1.343	0.49			

*AAD, anti-arrhythmic drug; BMI, body mass index; CBA, cryoballoon ablation; CI, confidence interval; HR, hazardratio; LA, left atrium; CHA2DS2-VASc, Congestive heart failure, Hypertension, Age ≥75 years (doubled), Diabetes mellitus, prior Stroke or TIA or thromboembolism (doubled), Vascular disease, Age 65 to 74 years, Sex category*.

* *CBG2, CBG3, or CBG4*.

*Bold values are referred statistically significant*.

### Durability of PVI

From 2007 to 2020, patients with symptomatic recurrences after CBA were treated according to the guidelines, and 268 (23.5%) of the 1,140 patients underwent a repeat ablation procedure because of symptomatic recurrences. The median time span from the initial ablation procedure to repeat ablation was 567.5 (275.5; 1191.5) days. In 96 (35.8%) of the patients, repeat ablation was performed after first-generation CBA. The majority of patients (172/268, 61.3%) underwent repeat AF ablation by means of radiofrequency catheter ablation guided by 3D-electroanatomical high-density mapping after initial advanced CBA (CBG2, CBG3, or CBG4).

Of the 268 patients with a clinical indication for repeat ablation, 133 (49.6%) demonstrated durable PVI of all veins. Patients pretreated with CBG1 demonstrated a significantly lower rate of complete PVI as compared with patients scheduled for repeat ablation after advanced CBA (18.8 vs. 66.4%, *p* < 0.001).

The percentage of PV with permanent disconnection at repeat ablation, in total, was 73.5%, after CBG1 the rate was lower (48.4% of PVs), and after advanced CBA the rate was highest (88% of PVs). Details are presented in Table 5.

**Table 5:** Pulmonary vein electrical reconnection at repeat ablation.

**Number of PV with reconnection**	**Total number of patients with repeat ablation *N* = 268**	**Number of patients with repeat ablation after first generation CBA *N* = 96**	Number of patients with repeat ablation after advanced CBA^*^N = 172
0	133 (49.6)	18 (18.8)	115 (66.4)
1	60 (22.4)	18 (18.8)	42 (24.3)
2	36 (13.4)	25 (26.0)	11 (6.4)
3	14 (5.2)	13 (13.5)	1 (0.57)
4	24 (9.0)	21 (21.9)	3 (1.7)
5	1 (0.4)	1 (1.0%)	0 (0)
**PV**	**% of PV with durable isolation**		
all PV	73.5	48.4	88.0
LSPV	73.2	47.3	88.1
LIPV	78.1	53.3	92.5
RIPV	73.6	45.7	89.6
RSPV	70.8	49.5	82.9
RMPV	45.5	42.9	50.0
LCPV	55.6	0.0	83.3

*Numbers with percentages*.

*PV, pulmonary vein; LCPV, left common PV; LIPV, left inferior PV; LSPV, left superior PV; RIPV, right inferior PV; RMPV, right middle PV; RSPV, right superior PV*.

* *CBG2, CBG3, or CBG4*.

## Discussion

This is the first and largest study that evaluates PVI by means of CBA as the general ablation strategy in all-comers for the initial ablation procedure in symptomatic persistent AF (excluding patients with a large LA diameter of > 60 mm). The main results of the study can be summarized as follows:

First, the PVI-only strategy is safe and effective in persistent AF with favorable rates of arrhythmia-free survival at 1 and 2 years after a single procedure.

Second, CBA in persistent AF creates durable PVI. At the time of the second procedure, 88% of PVs were isolated if one of the advanced CBA catheters was used.

Third, balloon technology, ablation protocols, lab management, and operator experience improved tremendously over time, and the procedure became safer, more efficacious, radiation-reduced, and faster.

Fourth, independent predictors of recurrence were female sex, LA diameter, and use of the first-generation of cryoballoon, which is no longer available.

### CBA as a Strategy for the Initial Procedure

In the early days of CBA for persistent AF, the first generation of cryoballoon was used in a double balloon procedure with the rationale of wide antral ablation in persistent AF in our center. The initial results served as a proof of concept for CBA in persistent AF (6). The rate of acute PVI increased from 99.1% (CBG1) to 100% (CBG4), *p* < 0.05. Although no difference was observed in the unadjusted log-rank comparison of the Kaplan-Meier plots for the outcome of first-generation vs. advanced CBA, the use of the advanced technology was an independent predictor for success after adjustment in the multivariate analysis. The high efficacy of the initial double balloon procedure with CBG1 was associated with longer left atrial dwelling times, higher amounts of radiation, more transient phrenic nerve injuries, and more pulmonary infections. Thus, the introduction of the more effective second-generation cryoballoon and its successors, with increased refrigerant flow rate, broader cooling-zone, and shorter catheter, led to better acute and chronic results despite the higher rate of comorbidities in the latter patient population. The implementation of smart ablation protocols with dosing strategies according to the individual time to electrical isolation of the PV contributed to reduced application times, which turns into shorter LA dwelling times without compromising the efficacy.

### Safety of CBA

Overall, CBA in the initial ablation procedure for persistent AF is safe. During more than 10 years of experience and 1,140 patients treated, no death was associated with the procedure and no atrio-esophageal fistula was occurred. The very low thromboembolic cerebrovascular complication rate could be explained by the lesions characteristics of cryoenergy in cardiac tissue, and minimal endocardial surface disruption (15). In addition, the rate of pericardial tamponade was also extremely low (1/1,140 [0.09%]) and might be attributed to the over-the-wire balloon platform, single-transseptal access approach, cryothermal energy source itself, experience of the operators, and use of echocardiographic (transesophageal echocardiography, TEE, or intracardiac echocardiography, ICE) guidance in all the cases. The observed rate of persistent phrenic nerve palsy was very low as compared with the literature (16), and the rate of transient phrenic nerve injury decreased significantly over time. Four main determinants were identified for the decline in transient PNP: experience of the operators, additional visualization of the diaphragmatic motion by ICE, abolition of the routine double balloon strategy and of the routine bonus application, and reduction in application times. Of note, neither the implementation of uninterrupted or shortly interrupted oral anticoagulation nor the implementation of propofol/morphine analgo-sedation had an impact on the safety profile of the procedure.

### Radiation Exposure

Radiation dose decreased significantly over time. From 2007 to 2020, all the procedures were performed on the same catheter lab x-ray system (Philips Allura cardiovascular X-ray system; Philips, Eindhoven, Netherlands) with presets for electrophysiological procedures. Dose reduction might be attributed to the practical implementation of the “As Low As Reasonably Achievable” (ALARA) principle in our center with shorter cine sequence acquisition, better collimation, reduced frame rates, and less angulation. In addition, the shorter LA dwelling times, improved steerable sheath, long-term learning curve of the operators, implementation of ICE in the lab, and significant lower BMI of the patients might have led to the reduction in radiation exposure.

### Predictors of Recurrence

Left atrial diameter was a strong independent predictor of recurrence. This result proves the observation of Reissmann et al. (17) and Akkaya et al. (18) on a larger population.

Female sex was an independent predictor for atrial arrhythmia recurrence in our study. Women with AF are older and have a higher prevalence of comorbidities compared with males (19). The relative risk of recurrences in women was 42% higher as compared with males (*p* = 0.004 in the multivariate analysis). In the 1STOP trial, with mainly patients with paroxysmal AF, female sex has also been reported to be an independent predictor for recurrence after CBA (20). Neither Reissmann nor Akkaya has found sex-related differences in CBA for persistent AF (17, 18). However, both of the studies were smaller, with 135 and 102 patients, respectively. Hypothetically, sex-related differences in the presence of fibrotic atrial myopathy or additional extra-PV trigger sites may explain our observations.

### Outcome and Durability of PVI

In the meta-analysis of Omran, freedom from AF following CBA in persistent AF was 68.9% after a mean FU of 16.7 ± 3 months (8). In the CRYO4PERSISTENT and the STOP Persistent AF trials, a 12-month success rate of 61 and 55%, respectively, was reported (7, 21). In our study, recurrences were observed in 45.6% of the patients, and the Kaplan–Meier estimates for arrhythmia-free survival was 81.8% at 1 year, and 61.7% at 2 years of follow-up. At 1-year follow-up, Ciconte et al. demonstrated that 60% of patients had stable sinus rhythm following persistent AF ablation with the novel cryoballoon (22). Differences in outcomes among centers might be influenced by differences in baseline characteristics, follow-up methods, operator experience, and definition of endpoints.

The favorable outcome with respect to arrhythmia-free survival at 1- and 2-years in our study might be associated with the durability of PVI following CBA in persistent AF. CBA is supposed to create less operator-dependent, reproducible, and comparable outcome results among centers, as reported by Providencia et al. (23). In paroxysmal AF, a rate of 1.4 ± 1.1 reconnected PV at repeat ablation has been demonstrated (24). Data on the rate of complete PVI in patients with persistent AF following initial sole cryoballoon PVI are scarce. All PVs were durably isolated in 66 of 81 (81.5%) patients with persistent AF following PVI plus posterior wall isolation using the cryoballoon (21). This manuscript includes the largest report on the durability of PVI following index CBA in persistent AF. Complete electrical PVI was present in 50% of the patients at repeat ablation. In the initial procedure, most of the patients were treated with one of the advanced, more effective cryoballoon catheters. Their higher efficacy was also demonstrated in our study by the significant increase in the durability of PVI from 18.8% after initial CBG1 ablation to 66.4% of the patients after initial advanced CBA. The fact that 88% of PVs in patients with symptomatic recurrences are still durably isolated after initial advanced CBA underscores the need for ablation strategies beyond PVI at the repeat procedure. The benefit of novel ultra-high density mapping systems in guiding ablation strategies in patients with recurrences and durable isolated PV following CBA has been reported recently (25).

### Limitations

This was a single-center observational study with inherent limitations accompanying this type of study design. The results were based on clinical visits and evaluation of symptoms, ECG, and Holter monitoring (1–7 days) during routine follow-up. Therefore, asymptomatic recurrence might have been missed. On the other hand, the recurrence rate might be overestimated, as typical symptoms in patients with paroxysmal AF were counted as recurrence if ECG documentation was difficult. No systematic, continuous monitoring with implantable devices was available.

In this trial, available cryoballoon ablation catheters (Medtronic Inc., Minneapolis, MN, United States) were used. It is unclear if these results are reproducible by novel cryoballoon systems.

## Conclusion

Pulmonary vein isolation in symptomatic persistent AF is a reasonable strategy in the initial ablation procedure for rhythm control. Moreover, CBA in this setting provides a safe and effective technique with low radiation exposure, short left atrial dwelling times, favorable outcomes, and high rates of durably isolated PVs after a single procedure. Our results underscore the need for ablation strategies beyond PVI for repeat AF ablation procedures, because PV reconnection is no longer a common finding with the currently available cryoballoon technology.

## Data Availability Statement

The datasets presented in this article are not readily available because the database was kept on the server of the Heart-Center Munich-Bogenhausen, Munich, Germany. The authors confirm that the data supporting the findings of this study are available within the article and/or its supplementary materials. Due to the nature of this research, participants of this study did not agree for their data to be shared publicly, so supporting raw data is not available. Requests to access the datasets should be directed to florian.straube@muenchen-klinik.de.

## Ethics Statement

The studies involving human participants were reviewed and approved by Bavarian State Camber of Physicians. The patients/participants provided their written informed consent to participate in this study.

## Author Contributions

FS analyzed the data, interpreted the results, and drafted the manuscript and figures. JP analyzed the data, was responsible for the statistics, drafted tables and figures, and interpreted the results. This study contains data from the doctoral thesis of AK, who contribute data acquisition, follow-up, and data analysis and interpretation. UD, EH, and FS designed the study protocol. FS, UD, LR, BB, SH, CT, UE, and MW contributed to patient recruitment and data acquisition. BB was responsible for data management. EH conducted the study as the head of the department, substantially contributed to the conception of the study, patient recruitment, data acquisition, and interpretation of the results. All authors substantially contributed to the study, critically revised the manuscript for important intellectual content, approved the manuscript, and agreed to be accountable for all aspects of the study.

## Funding

AK and JP received scholarships from the independent non-profit Zenner Foundation, Munich, Germany.

## Conflict of Interest

FS received honoraria for lectures from Medtronic and Bristol-Myers-Squibb outside the submitted study and educational support from Pfizer. UD reports honoraria for lectures from Medtronic Inc., outside the submitted study. SH participates in the EP fellowship from Boston Scientific, received educational support from Biotronik, Daiichi Sankyo, and honoraria for lectures from Bristol Myers Squibb outside the submitted study. EH was head of the department; the department received compensation for participation in clinical research trials outside the submitted study from Abbott, Bayer, Biotronik, Boehringer Ingelheim, Edwards, Elixier, Medtronic, and Stentys. The remaining authors declare that the research was conducted in the absence of any commercial or financial relationships that could be construed as a potential conflict of interest.

## Publisher's Note

All claims expressed in this article are solely those of the authors and do not necessarily represent those of their affiliated organizations, or those of the publisher, the editors and the reviewers. Any product that may be evaluated in this article, or claim that may be made by its manufacturer, is not guaranteed or endorsed by the publisher.
